# A new option for laparoscopic spleen-preserving distal pancreatectomy: three cases with splenic artery preservation and resection of the splenic vein

**DOI:** 10.1093/jscr/rjac088

**Published:** 2022-04-03

**Authors:** Masataka Okuno, Yasuhiro Shimizu, Yoshiki Senda, Seiji Natsume, Shoji Kawakatsu, Seiji Ito, Koji Komori, Tetsuya Abe, Kazunari Misawa, Yuichi Ito, Takashi Kinoshita, Eiji Higaki, Hironori Fujieda, Yusuke Sato, Akira Ouchi, Masato Nagino, Kazuo Hara

**Affiliations:** Department of Gastroenterological Surgery, Aichi Cancer Center Hospital, Aichi, Japan; Department of Gastroenterological Surgery, Aichi Cancer Center Hospital, Aichi, Japan; Department of Gastroenterological Surgery, Aichi Cancer Center Hospital, Aichi, Japan; Department of Gastroenterological Surgery, Aichi Cancer Center Hospital, Aichi, Japan; Department of Gastroenterological Surgery, Aichi Cancer Center Hospital, Aichi, Japan; Department of Gastroenterological Surgery, Aichi Cancer Center Hospital, Aichi, Japan; Department of Gastroenterological Surgery, Aichi Cancer Center Hospital, Aichi, Japan; Department of Gastroenterological Surgery, Aichi Cancer Center Hospital, Aichi, Japan; Department of Gastroenterological Surgery, Aichi Cancer Center Hospital, Aichi, Japan; Department of Gastroenterological Surgery, Aichi Cancer Center Hospital, Aichi, Japan; Department of Gastroenterological Surgery, Aichi Cancer Center Hospital, Aichi, Japan; Department of Gastroenterological Surgery, Aichi Cancer Center Hospital, Aichi, Japan; Department of Gastroenterological Surgery, Aichi Cancer Center Hospital, Aichi, Japan; Department of Gastroenterological Surgery, Aichi Cancer Center Hospital, Aichi, Japan; Department of Gastroenterological Surgery, Aichi Cancer Center Hospital, Aichi, Japan; Department of Gastroenterological Surgery, Aichi Cancer Center Hospital, Aichi, Japan; Department of Gastroenterology, Aichi Cancer Center Hospital, Aichi, Japan

## Abstract

There are two techniques for a spleen-preserving distal pancreatectomy (SPDP): SPDP with splenic vessel preservation, and SPDP with splenic vessel resection. In some cases, although the splenic artery (SpA) can be preserved, the splenic vein (SpV) must be resected. We report the short- and long-term outcomes of three patients who underwent a new technique of laparoscopic SPDP with SpA preservation and SpV resection (SPDP-VRes). A grade B pancreatic fistula, which occurred in two patients, was successfully treated with drainage tube management. In all cases, the omental branches of the left gastroepiploic vein functioned as a drainage vein, and there was no splenomegaly, thrombocytopenia, or varix formation during the follow-up period (19 months to 5 years). Patients undergoing laparoscopic SPDP-VRes had no severe complications during the follow-up period; preserving the left omental branch is a key to this procedure. Laparoscopic SPDP-VRes might be a useful treatment option for patients undergoing SPDP.

## INTRODUCTION

Spleen-preserving distal pancreatectomy (SPDP) has been performed in patients with benign or low-grade malignancies of the body and tail of the pancreas who do not require lymph node dissection. There are two variations of the SPDP: SPDP with splenic vessel preservation (SPDP-Pre) [1], and SPDP with splenic vessel resection (SPDP-Res, the so-called Warshaw technique) [2]. Advances in laparoscopic surgery have made it possible to perform complex cases as closed rather than open procedures [3–6]. Although we have attempted to perform laparoscopic SPDP-Pre, dissection between the splenic vessels and pancreatic parenchyma can be difficult because of tumor size, inflammation and/or adhesions. In such cases, we have changed laparoscopic SPDP-Pre to laparoscopic SPDP-Res, or occasionally to laparoscopic SPDP with splenic artery (SpA) preservation and splenic vein (SpV) resection (SPDP-VRes). Herein, we report the short- and long-term outcomes of laparoscopic SPDP-VRes in three cases**.**

## LAPAROSCOPIC SPDP-VRES SURGICAL TECHNIQUES

**Figure 1:**
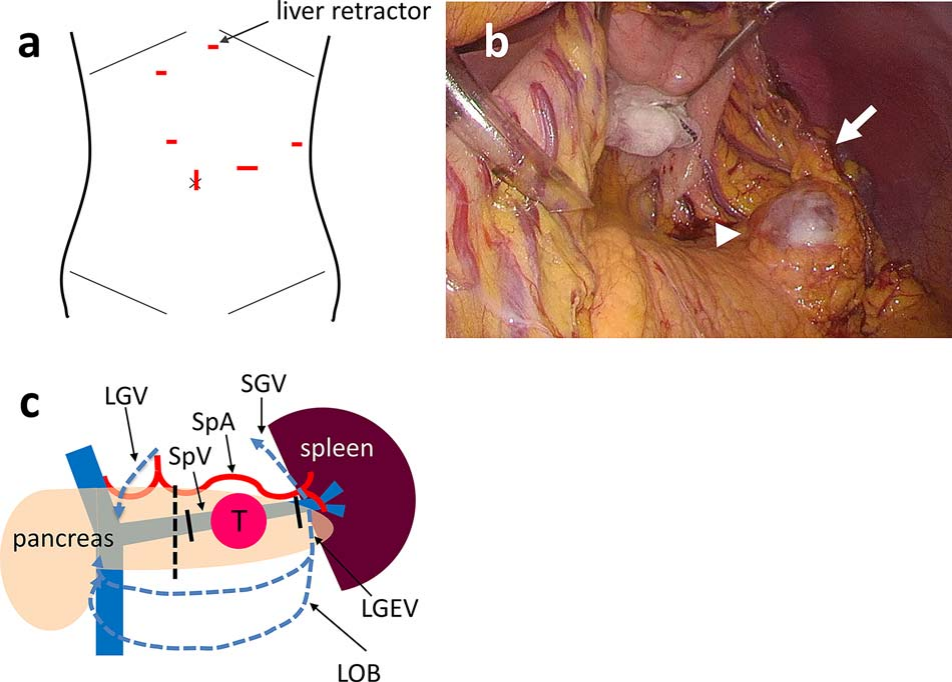
(**a**) One transumbilical access port is created for videolaparoscopy and working trocars are inserted into four other ports. A Nathanson hook liver retractor is placed in the subxiphoid location (arrow). (**b**) Intraoperative photograph of Case 3 showing that the stomach and lateral segment of the liver are retracted by the retractor. The cystic tumor (arrowhead) is exposed optimally even if the omental branches of the left gastroepiploic vessels remain (arrow). (**c**) Schema of laparoscopic SPDP-VRes. The pancreas is divided at the dissection line (dotted line). The splenic vein is divided at the proximal side of tumor and the pancreatic tail (solid line), preserving the confluence of the SGV, the LGEV and the LOB. The three drainage routes from the spleen are indicated by the blue dotted arrows: the arcade of the GEV; the arcade of the omental vein and the SGV to the LGV via the stomach wall. PV: portal vein, SMV: superior mesenteric vein, SpV: splenic vein, SpA: splenic artery, T: tumor.

The main elements of laparoscopic SPDP-VRes surgical procedures are as follows. The patient is placed in the supine position with the arms extended laterally with the legs apart. One transumbilical access port was used for videolaparoscopy and working trocars were inserted into four additional ports (Fig. 1a). A Nathanson liver retractor is placed in the subxiphoid location, which retracts the stomach and left lateral segment of the liver to the right in the cranial direction. The greater omentum is dissected along the distal aspect of the gastroepiploic vessels using a LigaSure™ Vessel Sealing System (Medtronic, Dublin, Ireland) and the lesser sac is entered. The left gastroepiploic, short gastric and omental branches of the left gastroepiploic vessels are carefully preserved to maintain the blood supply to the spleen and secure the drainage routes. Because the stomach and lateral segment of the liver are retracted, the pancreatic tail is optimally exposed, even if the omental branches of the left gastroepiploic vessels remain (Fig. 1b). The SpA and SpV are identified and taped near the dissection line. The pancreas is divided slowly using a stapler device at the line. The SpA is removed from the pancreas from the dissection line toward the spleen, while the SpV is clipped and divided at the proximal portion where SpV cannot be separated from the pancreatic parenchyma. To secure the drainage routes from the spleen, the SpV is identified and divided at the splenic hilum to preserve the confluences of the short gastric and left gastroepiploic veins (LGEV) and the omental branches (Fig. 1c). Closed drains are placed in the pancreas stump and left subphrenic space.

## CASE SERIES (**TABLE 1**)

**Table 1:** Clinical features of three cases who underwent laparoscopic SPDP-VRes

Case	Age, years	Gender	Diagnosis	Reason for resection of the SpV	Operative time	Blood loss, ml	Post-operative hospital stay, days	Major venous return routes from the spleen to SMV/PV	Platelet level, x10^4^/ μL		Spleen volume, cm^3^		Long-term complications
									Pre- operation	Post- discharge	Pre- operation	Post- discharge	
1	31	F	SPN	Close to the tumor	254 min	Little	35	LOB-ARCV SGV-Stomach-LGV GEV	27.1	17.7	195	144	None
2	46	F	pNET	Adhesion	267 min	20	37	LOB-MCV SGV-Stomach-LGV	26.3	17.0	126	145	None
3	38	F	MCN	Close to the tumor	241 min	Little	9	LOB-MCV-SpV	15.4	13.0	133	185	None

SPDP-VRes: spleen-preserving distal pancreatectomy with splenic artery preservation and splenic vein resection, F: female, SPN: solid pseudopapillary neoplasm, pNET: pancreatic neuroendocrine tumor, MCN: mucinous cystic neoplasm, SpV: splenic vein, SMV: superior mesenteric vein, PV: portal vein, LOB: left omental branch, ARCV: accessory right colic vein, SGV: short gastric vein, LGV: left gastric vein, GEV: gastroepiploic vein, MCV: middle colic vein.

### Case 1

**Figure 2:**
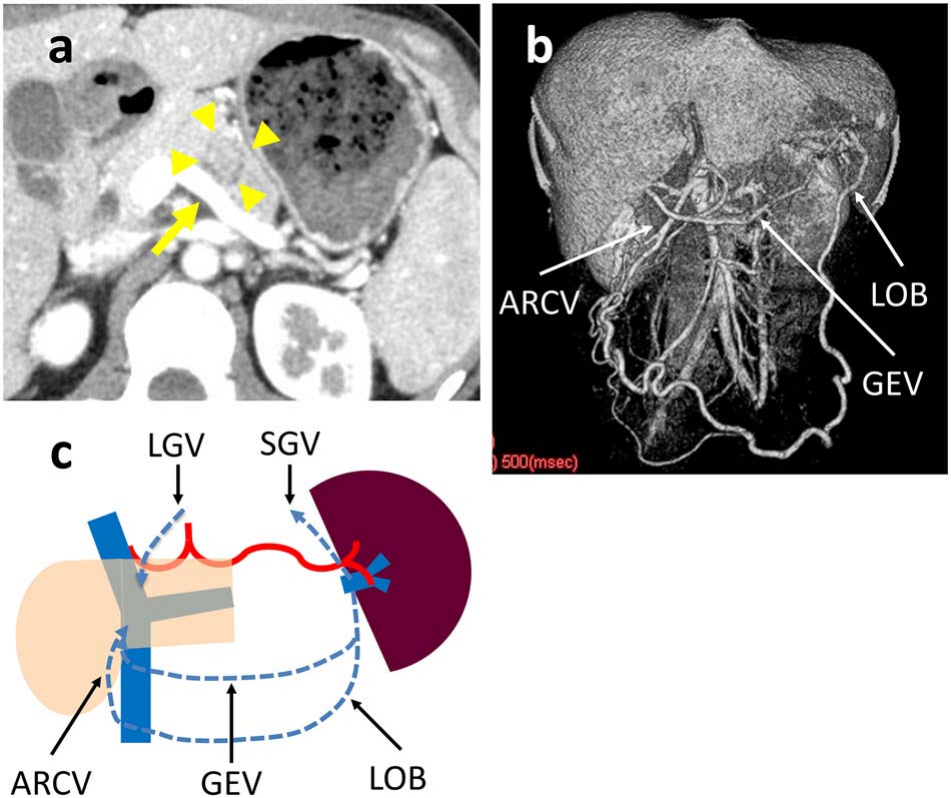
(**a**) Contrast-enhanced CT shows a 25-mm tumor in the pancreatic body (arrowhead) that is in wide contact with the splenic vein (arrow). (**b**) 3D reconstruction image shows a dilated GEV and the arcade that is formed by the LOB and ARCV. (**c**) Schema of venous return in Case 1. The blood flow from the spleen flows into the portal vein through three main routes: LOB to ARCV; the SGV to the LGV via the stomach wall; and GEV.

A 31-year-old woman was diagnosed with a solid pseudopapillary neoplasm (SPN) based on endoscopic ultrasound-guided fine needle aspiration (EUS-FNA) cytology. Contrast-enhanced computed tomography (CT) showed a 25-mm tumor with calcifications in the pancreatic body that widely contacted the SpV, but was not close to the SpA (Fig. 2a). It was difficult to surgically separate the SpV from the pancreatic parenchyma around the tumor at the pancreatic body; therefore, the SpV was divided at the proximal side of the tumor and the pancreatic tail. The operative time was 254 min and the blood loss was negligible. The patient developed a grade B pancreatic fistula [7], which required drainage tube management. The patient was discharged on postoperative Day 35. The histologic diagnosis was a SPN with infiltrative growth to the pancreatic parenchyma and contact with the SpV wall. The surgical margin was negative for tumor cells. A postoperative CT scan revealed that the omental branch of the LGEV, left gastric vein (LGV) and GEV were dilated. There were no gastric varices and no gastric wall enhancement (Fig. 2b). There was no SpA stenosis. Blood flowed from the spleen into the superior mesenteric vein (SMV) and portal vein (PV) via three main routes: the omental arcade to the accessory right colic vein (ARCV); the short gastric vein (SGV) to the LGV via stomach wall and the gastroepiploic arcade (Fig. 2c). The preoperative and post-discharge (47 postoperative days [PODs]) platelet levels were 27.1 and 17.7 x 10^4^/μL, respectively. The pre- and post-operative (8 postoperative months [POMs]) spleen volumes measured by CT scan were 195 and 144 cm^3^, respectively. The patient remains healthy without a recurrence 5 years postoperatively.

### Case 2

**Figure 3:**
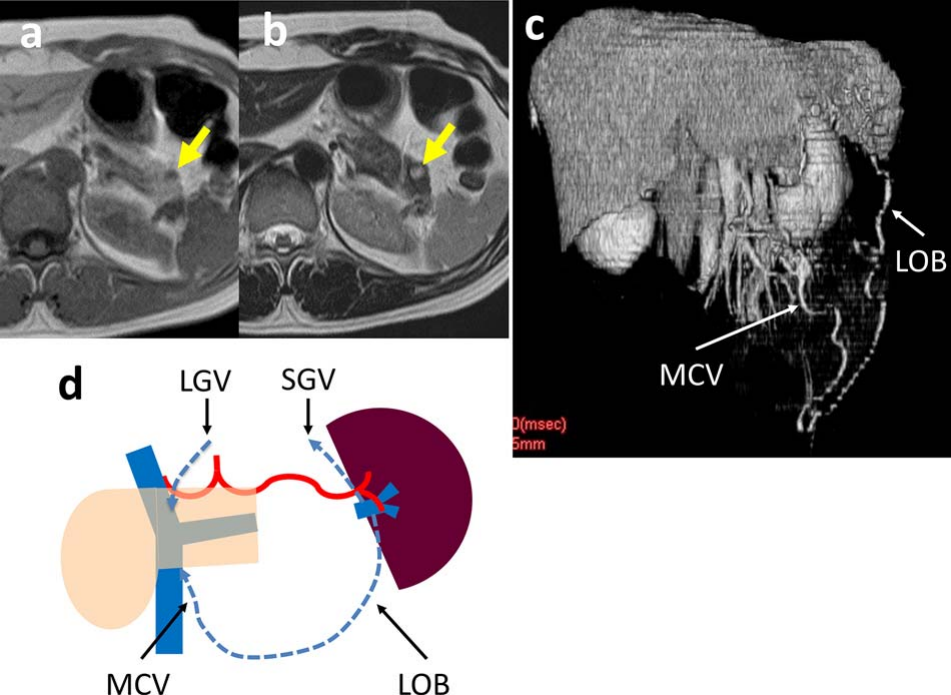
(**a**, **b**) Contrast-enhanced MRI shows a 5-mm tumor in the pancreatic tail (arrow) that has a low-signal intensity on T1-weighted images and a high-signal intensity on T2-weighted images. (**c**) 3D reconstruction image shows an arcade formed by the dilated LOB and MCV. (**d**) Schema of venous return in Case 2. The blood flows from the spleen into the SMV/PV through two main routes: LOB to MCV; and the SGV to the LGV via the stomach wall.

A 46-year-old woman was shown to have hypoglycemia during a medical examination. She presented to our hospital for evaluation of worsening hypoglycemic symptoms. A CT scan of the abdomen did not reveal any abnormalities. Contrast-enhanced magnetic resonance imaging (MRI) showed a 5-mm tumor in the pancreatic tail which had a low-signal intensity on T1-weighted images and a high-signal intensity on T2-weighted images (Fig. 3a and b). EUS-FNA cytology indicated a pancreatic neuroendocrine tumor (pNET). An insulinoma was highly suspected and we performed a laparoscopic spleen-preserving procedure. There was a scar on the ventral surface around the tumor, which was thought to be caused by inflammation after the EUS-FNA. Although the SpA could be preserved because the SpA was not buried in the pancreatic parenchyma, it was difficult to separate the SpV from the pancreatic parenchyma around the scar because of adhesions and bleeding. The operative time was 267 min and the estimated blood loss was 20 cc. The patient developed a grade B pancreatic fistula [7], which required drainage tube management. The patient was discharged on Day 37 postoperatively. The histologic diagnosis was a pNET (10 mm in diameter) that did not contact the SpV and the surgical margins were negative for tumor cells. A postoperative CT scan revealed marked dilation of the omental branch of the LGEV (Fig. 3c). There was no SpA stenosis. The blood flowed from the spleen into the SMV via the left omental branch (LOB) through the middle colon vein. Although there was increased venous blood flow into the gastric fundus wall via the SGV and dilation of the LGV, no varices were observed. No right and LGEV arcade formed (Fig. 3d). The preoperative and post-discharge (47 PODs) platelet levels were 26.3 and 17.0 x 10^4^/μL, respectively. Pre- and post-operative (5 POMs) spleen volumes were 126 and 145 cm^3^, respectively. The hypoglycemic symptoms improved and she has been alive without a recurrence 4 years postoperatively.

### Case 3

**Figure 4:**
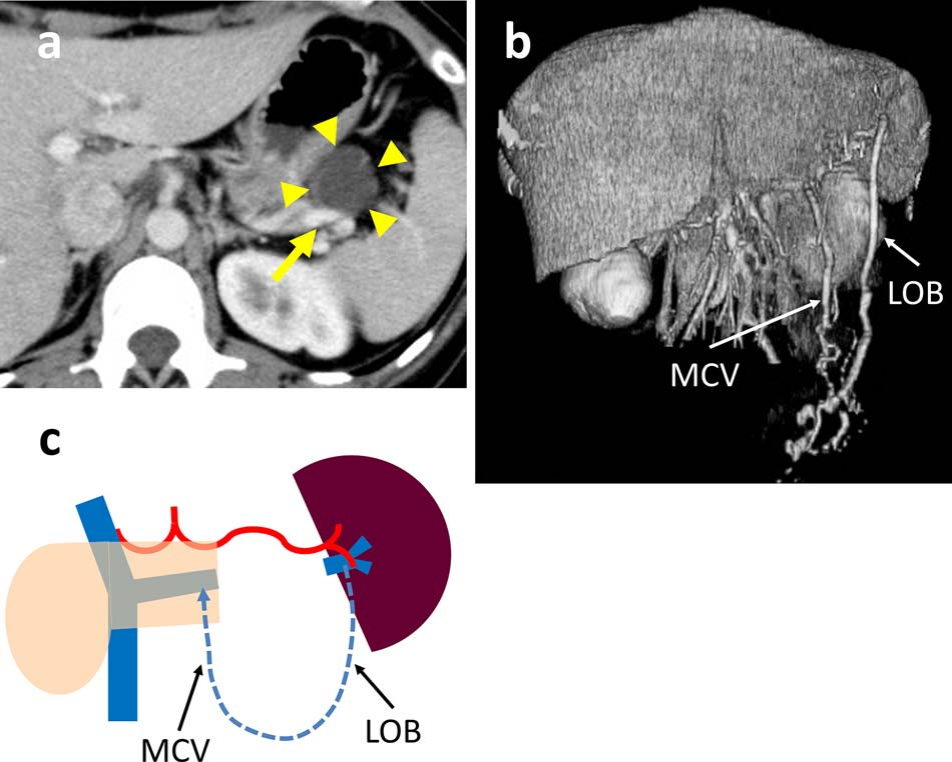
(**a**) Contrast-enhanced CT shows a 35-mm cystic tumor in the pancreatic tail (arrowhead) which is in contact with the splenic vein (SpV) (arrow). (**b**) 3D reconstruction image shows the arcade formed by the dilated LOB and MCV. (**c**) Schema of venous return in Case 3. The blood flows from the spleen into the PV via the LOB to the MCV and SpV.

A 38-year-old woman was diagnosed with a mucinous cystic neoplasm (MCN) based on imaging studies. A CT scan showed a 35-mm cystic tumor in the pancreatic tail which was in contact with the SpV and close to the SpA (Fig. 4a). Although the SpA could be surgically dissected and preserved, dissecting the SpV from the cystic tumor was difficult due to dense adhesions, which could result in cyst fluid leakage and residual tumor. The operative time was 241 min and the blood loss was minimal. The postoperative course was uneventful and the patient was discharged on day 9 after surgery. The histologic diagnosis was a MCN; there was no border between the SpV and cyst wall due to fibrosis. The surgical margins were negative for tumor cells. A postoperative CT scan revealed marked dilation of the omental branch of the LGEV without gastric varices (Fig. 4b). There was no SpA stenosis. The blood flowed from the spleen into the SMV via the LOB through the middle colon and splenic veins. No right and LGEV arcade formed (Fig. 4c). The preoperative and post-discharge (26 PODs) platelet levels were 15.4 and 13.0 x 10^4^/μL, respectively. The pre- and post-operative (7 POMs) spleen volumes were 133 and 185 cm^3^, respectively. The patient remains healthy without a recurrence 19 months postoperatively.

## DISCUSSION

Reports on laparoscopic SPDP have been increasing [3–6] because of advances in laparoscopic surgery and recognition of the importance in preserving the spleen. The spleen has important roles in the immune system and in removing blood cells. In addition, resection of the spleen leads to overwhelming post-splenectomy infections (OPSIs). Although the incidence of OPSIs in adults is low, sepsis mortality rates as high as 50% have been reported [8].

Splenic vessel preservation may be the optimal way to preserve blood flow to the spleen, but the SpV must be resected during this process in some cases. The SpA usually passes through the cranial portion of the pancreas and there are a few branches from the SpA. Therefore, it is relatively easy to divide the pancreas and the SpA. Because the SpV usually passes through the groove behind the pancreatic body and tail and there are many small branches into the SpV, the SpV is relatively difficult to divide from the pancreas. Moreover, if there is pancreatitis and adhesions to the SpV, it is more difficult to divide the vein and pancreas without injury or bleeding. In addition, there are other reasons for surgical difficulty; specifically, tumors close to the SpV are a risk for residual lesions, perforation of the cystic wall and tumors that are too large to secure the field of view. In such cases, SPDP-Res or conventional distal pancreatectomy with splenectomy is generally indicated.

Because SPDP-Res is a relatively simple procedure compared with the SPDP-Pre, the SPDP-Res has a shorter operative time and less blood loss [9]. Splenic infarction and abscess of the conserved spleen are more frequent in SPDP-Res than SPDP-Pre [4, 5, 9, 10]. This finding may be due to insufficient blood flow from the short gastric and gastroepiploic arteries, which are usually preserved in the SPDP-Res. To avoid ischemia of the spleen, we have reported a new technique, the SPDP-VRes, in which the SpA is preserved and the SpV is resected. Indeed, there are no reports on this procedure.

Congestion of the spleen and the development of gastric varices is a concern when performing a SPDP-VRes. Blocking SpV flow and preserving the SpA is similar to a pancreaticoduodenectomy with combined PV resection for pancreatic cancer invading the PV/SMV confluence, which results in left-sided portal hypertension [11, 12]. To prevent left-sided portal hypertension after a pancreaticoduodenectomy with resection of the PV/SMV confluence, preserving multiple drainage veins as much as possible is considered efficacious [11, 13, 14]. When performing a SPDP-VRes, we always preserve the omental branches of the LGEV as an essential drainage vein, as well as the short gastric and LGEVs that are normally preserved in the SPDP. The LOB forms the arcade in the lower omentum with the right omental branch (the venous arch of Barkow) [15], and blood flows through the gastrocolic trunk or the middle colon vein to the PV. The preserved LGEV can also serve as collateral circulation, but in some cases an arcade with the right GEV is not formed. In the three cases presented herein, the GEV arcade was confirmed in only one case (Case 1) on postoperative examination. The preserved LOB functioned as an important drainage vein and there were no complications, such as thrombocytopenia, splenomegaly and varix formation, in all three cases who underwent SPDP-VRes during a relatively long-term follow-up.

In the previous reports of SPDP-Res and SPDP-Pre, the left omental vessels and the splenocolic ligament are divided [1, 2]. This certainly provides a better view of the splenic hilum, but it sacrifices the drainage vein of the left omental vein. Even if the left omental vein is preserved, the field of view of the splenic hilum can be obtained by properly retracting the stomach and left lateral segment of the liver to the right in the cranial direction. Preserving the LOBs is a key point in all SPDP procedures, especially in the SPDP-VRes because the LOBs function as an essential drainage vein.

## CONCLUSIONS

SPDP-VRes may be a useful treatment option for patients who have tried to undergo SPDP-Pre. There were no serious complications during short- and long-term follow-up. It is important to preserve the omental branches of the LGEV to avoid spleen congestion.
